# Observation of linear and quadratic magnetic field-dependence of magneto-photocurrents in InAs/GaSb superlattice

**DOI:** 10.1186/1556-276X-9-279

**Published:** 2014-05-31

**Authors:** Yuan Li, Yu Liu, Chongyun Jiang, Laipan Zhu, Xudong Qin, Hansong Gao, Wenquan Ma, Xiaolu Guo, Yanhua Zhang, Yonghai Chen

**Affiliations:** 1 Key Laboratory of Semiconductor Materials Science, Institute of Semiconductors, Chinese Academy of Sciences, No.A35, Tshinghua East Road, Haidian District, Beijing 100083, China; 2 Laboratory of Nano-Optoelectronics, Institute of Semiconductors, Chinese Academy of Sciences, No.A35, Tshinghua East Road, Haidian District, Beijing 100083, China

**Keywords:** Magneto-photocurrents, Asymmetric excitation and relaxation, Spin-orbit interaction

## Abstract

We experimentally studied the magneto-photocurrents generated by direct interband transition in InAs/GaSb type II superlattice. By varying the magnetic field direction, we observed that an in-plane magnetic field induces a photocurrent linearly proportional to the magnetic field; however, a magnetic field tilted to the sample plane induces a photocurrent presenting quadratic magnetic field dependence. The magneto-photocurrents in both conditions are insensitive to the polarization state of the incident light. Theoretical models involving excitation, relaxation and Hall effect are utilized to explain the experimental results.

## Background

**Figure 1:**
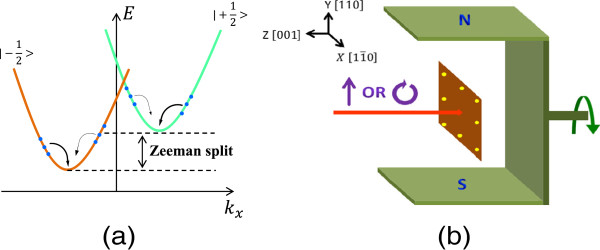
**Schematic diagram (a) of nonequilibrium electrons which occupy two spin-splitting energy bands and experimental setup diagram (b). (a)** An in-plane magnetic field perpendicular to *k*_*x*_ is applied to induce the Zeeman split energy *ΔE*=*g*^∗^*μ*_*B*_*B*. The blue dots stand for photo-excited nonequilibrium electrons. Curving arrows show the electron relaxation process. The thicker arrows mean the higher relaxation rate. **(b)** The magnetic field is rotated in the *x-y* plane.

Recently, spin-polarized transport has been a main topic of spintronics. Optical injection has been widely used to generate a spin current [1,2]. In low-dimensional semiconductor structures which possess structure inversion asymmetry (SIA) or bulk inversion asymmetry (BIA), the spin-orbit interaction (SOI) lifts the spin degeneracy in k space and leads to a linear spin splitting [3]. A normally incident linearly polarized or unpolarized light can excite identical amount of nonequilibrium carriers with opposite spins and velocities to the spin-splitting subbands, leading to a spin photocurrent, accompanied by no electric current. Direct detection of the spin current is difficult for the absence of net current and polarization. However, as shown in Figure 1a, the symmetric distribution of electrons can be broken by the Zeeman splitting caused by a magnetic field, then the magneto-photocurrent effect (MPE) occurs [4]. The spin-polarized magneto-photocurrent provides an effective approach to research the spin current.

MPE has been observed in InGaAs/InAlAs two-dimensional electron gas, GaAs/AlGaAs quantum well, graphene and so on [5-7]. By comparison, the InAs/GaSb type II supperlattice has some advantages in investigating spin transport and fabricating spintronic devices for its properties of large SOI in InAs and GaSb, relatively high carrier mobility in InAs and peculiar energy band structure [8,9]. Previously, the InAs/GaSb type II superlattice has been extensively researched as an infrared detector. The studies have been mainly focused on carrier recombination, interface properties, tailoring of energy bands and so on [10-17]. The zero-field spin splitting has also been observed in InAs/GaSb quantum wells by Shubnikov-de-Haas oscillation [18], while the investigations on the magneto-photo effect is seldom concerned. In the present paper, we investigate the MPE in the InAs/GaSb type II supperlattice. Unlike the previous researches of the magnetic field strength dependence of the photocurrents, we mainly focus on the magnetic field direction dependence of the photocurrents in this structure. By varying magnetic field direction in or out of the sample plane, we observed linear and quadratic magnetic field dependence of the photocurrents, respectively. More information about excitation and relaxation of electrons in this structure were obtained from the experiments.

## Methods

The InAs/GaSb superlattice was fabricated by molecular beam epitaxy technique on semi-insulating (001)-oriented GaAs substrate. The 500-nm GaAs and 1,000-nm GaSb buffers were deposited on the substrate to relieve the lattice mismatch. Then an InAs/GaSb superlattice of 155 periods was deposited. The monolayer thicknesses of InAs and GaSb are 3.85 and 2.60 nm, respectively. The sample was not intentionally doped. The energy gap of this structure calculated by the k ·p theory is 129.5 meV. The standard Hall measurement demonstrates that the sample is n-type at room temperature, i.e. electrons are the main carriers contributing to transport. Since in the n-type superlattice spin relaxation time and lifetime of holes are much shorter than those of electrons, we neglect the contribution of holes to the magneto-photocurrents. Four pairs of ohmic contact electrodes which are parallel to [1$\bar{1}$0], [110], [100] and [010] crystallographic directions were equidistantly made on the edges.

The experimental setup is shown in Figure 1b. A linearly polarized 1,064-nm laser normally irradiated on the center of the sample to excite direct interband transition of electrons. Hence, the circular photogalvanic effect and linear photogalvanic effect [3] are forbidden in this C _2*v*_ symmetry structure for the normal incidence case. A permanent magnet was used to generate magnetic field which can be along arbitrary direction in the sample plane. The investigation of photogalvanic effect was carried out at room temperature by rotating the magnetic field. The data were collected by a standard lock-in amplification technique. Specifically, the laser power was about 63 mW, the light spot diameter was 1.2 mm and the permanent magnet strength was 0.1 T. Besides, we choose x, y and z to be along [1$\bar{1}$0], [110] and [001] crystallographic directions, respectively.

## Results and discussion

### In-plane magnetic field-dependent MPE

**Figure 2:**
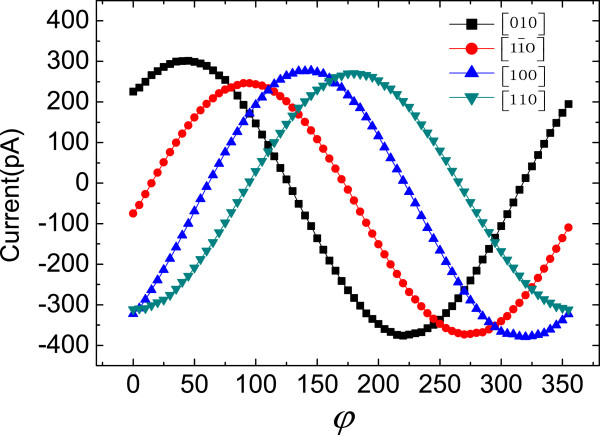
**The currents in [010], [1**$\bar{1}$**0], [100] and [110] crystallographic directions when the linearly polarized direction of the incident light is along [110] crystallographic direction.***φ* is the angle between the magnetic field direction and [1$\bar{1}$0] crystallographic direction.

As shown in Figure 2, by rotating the magnetic field in the *x-y* plane, the MPE currents in [1$\bar{1}$0], [110], [100] and [010] crystallographic directions were detected. The current, as a function of *φ*, can be simulated by the combination of sin*φ* and cos*φ* no matter which pair of electrodes are chosen. They reach the maximum when the magnetic field is perpendicular to the detected direction and the minimum when the magnetic field is paralleled to the detected direction.

By extracting the peak-to-peak values of the currents (*J*_pp_) in four crystallographic directions, we observed that *J*_pp_ in the [100] and [010] crystallographic directions are larger than that in the [1$\bar{1}$0] and [110] directions. Merely considering the SOI-induced anisotropic splitting of the energy bands (see [3]) seems unable to explain this experimental result. Actually, the total photocurrents(described by *J*_pp_) are decided by both SOI and Zeeman splitting. The SOI generates the spin-dependent asymmetric transition matrix elements and scattering matrix elements in excitation and relaxation processes, respectively, which lead to the asymmetric distribution of electrons in each spin-splitting subband. The Zeeman splitting transforms the net spin currents to charge currents. Hence, the photocurrents are proportional to the Zeeman split energy and then the electron effective g-factor g ^∗^. In view of this, there are no common anion and cation in the InAs/GaSb superlattice interface; this structure belongs to the C _2*v*_ symmetry. Hence, g ^∗^ presents in-plane anisotropy when the magnetic field is in different crystallographic directions [19]. We speculated that the co-effect of the anisotropic SOI and g ^∗^ make *J*_pp_ in the [100] and [010] crystallographic directions larger.

For detailed analysis, the magnetic field direction dependence of the photocurrents can be well described by [20]

(1) $$\begin{array}{ll} j_{x}= & S_{1}B_{y}I+S_{2}B_{y}\left({\left|e_{x}\right|}^{2}-{\left|e_{y}\right|}^{2}\right)I\\ & +S_{3}B_{x}\left(e_{x}{e_{y}}^{*}+e_{y}{e_{x}}^{*}\right)I+S_{4}B_{x}P_{\text{circ}}I+C_{1}. \end{array}$$

(2) $$\begin{array}{lll} j_{y} & ={S_{1}}^{\prime }B_{x}I+{S_{2}}^{\prime }B_{x}\left({\left|e_{x}\right|}^{2}-{\left|e_{y}\right|}^{2}\right)I\; & \;\\ & \;+{S_{3}}^{\prime }B_{y}\left(e_{x}{e_{y}}^{*}+e_{y}{e_{x}}^{*}\right)I+{S_{4}}^{\prime }B_{y}P_{\text{circ}}I+C_{2}.\; \end{array}$$

The first terms on the right-hand side of Equations 1 and 2 (described by *S*_1_ and *S*_1_^′^) yield currents independent of the radiation polarization. The terms described by parameters *S*_2_, *S*_2_^′^ and *S*_3_, *S*_3_^′^ yield radiation linear polarization related currents proportional to |*e*_*x*_|^2^−|*e*_*y*_|^2^= cos(2*α*) and *e*_*x*_*e*_*y*_^∗^+*e*_*y*_*e*_*x*_^∗^= sin(2*α*), respectively, where *α* is the angle between the plane of linear polarization and the *x*-axis. The terms proportional to the circularly polarized degree *P*_circ_ (described by *S*_4_ and *S*_4_^′^) vanish for linearly polarized light excitation. *I* is the intensity of the incident light, it can be determined by light power per unit area of light spot. *B*_*x*_=*B*_0_ cos(*φ*), *B*_*y*_=*B*_0_ sin(*φ*), *B*_0_ = 0.1 T. *φ* is the angle between the magnetic field direction and [1$\bar{1}$0] crystallographic direction. *C*_1_ and *C*_2_ are background currents induced by the slight reduction of symmetry of the superlattice. The reduced symmetry is due to slight misorientation of substrate or presence of strain in the structure [21]. The background currents are independent of the magnetic field direction and polarization state of the incident light. So these currents will not affect the discussion of magneto-photocurrents. To describe the magneto-photocurrents in [100] and [010] crystallographic directions, we should change the coordinate system to *x*^′^∥ [100] and *y*^′^∥ [010]. Then the photocurrents can be described by [20]

(3) $$\begin{array}{lll} j_{x^{\prime }} & ={S_{1}}^{+}B_{x^{\prime }}I+{S_{1}}^{-}B_{y^{\prime }}I-\left({S_{2}}^{+}B_{x^{\prime }}+{S_{2}}^{-}B_{y^{\prime }}\right)\; & \;\\ & \;\times \left(e_{x^{\prime }}{e_{y^{\prime }}}^{*}+e_{y^{\prime }}{e_{x^{\prime }}}^{*}\right)I+\left({S_{3}}^{+}B_{x^{\prime }}-{S_{3}}^{-}B_{y^{\prime }}\right)\; & \;\\ & \;\times \left({\left|e_{x^{\prime }}\right|}^{2}\;-\;{\left|e_{y^{\prime }}\right|}^{2}\right)I+\left({S_{4}}^{+}B_{x^{\prime }}\;-\;{S_{4}}^{-}B_{y^{\prime }}\right)P_{\text{circ}}I\;+{C_{1}}^{\prime }.\; \end{array}$$

(4) $$\begin{array}{lll} j_{y^{\prime }} & =-{S_{1}}^{-}B_{x^{\prime }}I-{S_{1}}^{+}B_{y^{\prime }}I+\left({S_{2}}^{-}B_{x^{\prime }}+{S_{2}}^{+}B_{y^{\prime }}\right)\; & \;\\ & \;\times \left(e_{x^{\prime }}{e_{y^{\prime }}}^{*}+e_{y^{\prime }}{e_{x^{\prime }}}^{*}\right)I+\left(-{S_{3}}^{-}B_{x^{\prime }}+{S_{3}}^{+}B_{y^{\prime }}\right)\; & \;\\ & \;\times \left({\left|e_{x^{\prime }}\right|}^{2}-{\left|e_{y^{\prime }}\right|}^{2}\right)I+\left(-{S_{4}}^{-}B_{x^{\prime }}+{S_{4}}^{+}B_{y^{\prime }}\right)P_{\text{circ}}I\; & \;\\ & \;+{C_{2}}^{\prime }.\; \end{array}$$

Similar to the parameters in Equations 1 and 2, *S*_1_^±^ denote radiation polarization unrelated currents. Linearly and circularly polarized light related currents are described by *S*_2_^±^, *S*_3_^±^ and *S*_4_^±^, respectively. *C*_1_^′^ and *C*_2_^′^ are background currents.

To fit the photocurrent curves when the linearly polarized direction of the incident light is along [1$\bar{1}$0], [110], [100] and [010] crystallographic directions, respectively, we find that parameters *S*_1_, *S*_1_^′^ and *S*_1_^−^ are considerably larger than parameters *S*_2_, *S*_2_^′^, *S*_2_^±^, *S*_3_, *S*_3_^′^ and *S*_3_^±^. The detailed fitting results of the parameters are listed in Table 1. This reveals that polarization independent currents are dominant in total magneto-photocurrents. Furthermore, we found that the parameters *S*_1_ and *S*_1_^′^ are slightly smaller than *S*_1_^−^. The polarization-independent currents present anisotropy of crystallographic directions. The parameters of linearly polarized light-induced photocurrents are in the same order of magnitude except the *S*_3_ is larger.

**Table 1:** Fitting results of the parameters

	**Value**
*S*_1_	5.535
*S*_2_	−0.015
*S*_3_	0.383
*S*_1_^′^	−5.241
*S*_2_^′^	−0.003
*S*_3_^′^	0.018
*S*_1_^+^	0.269
*S*_1_^−^	−6.093
*S*_2_^+^	−0.016
*S*_2_^−^	−0.015
*S*_3_^+^	0.002
*S*_3_^−^	−0.018

Units: ${\text{10}}^{-\text{14}}\left[\frac{{\text{A}}^{\text{2}}{\text{s}}^{\text{5}}}{{\text{kg}}^{\text{2}}}\right]$.

From the microscopic point of view, the electric photocurrent density can be calculated by summing the velocities of the photo-excited carriers. The magneto-photocurrent in *μ* direction (*μ*=*x*,*y*) can be described by [5,22]

(5) $$\begin{array}{l} j_{\mu }=-e\underset{c,v,\vec{k}}{\sum }\rho_{\text{cv},\vec{k}}v_{\text{cv},\vec{k}}^{\mu } \end{array}$$

e is the electron charge. $v_{\text{cv},\vec{k}}^{\mu }$ denotes the electron velocity along *μ* direction. In the excitation process, $\rho_{\text{cv},\vec{k}}$ is the steady-state nonequilibrium photo-excited electron density in Zeeman-splitting conduction bands. It can be described by Equation 6 for the linearly polarized radiation.

(6) $$\begin{array}{ll} \rho_{\text{cv},\vec{k}}= & \rho_{\text{cv},\phi }^{\left(0\right)}+\rho_{\text{cv},\phi }^{\left(1\right)}\cos(2\phi )\cos(2\alpha )\\ & +\rho_{\text{cv},\phi }^{\left(2\right)}\sin(2\phi )\sin(2\alpha ) \end{array}$$

*ϕ* is the angle between the wave vector $\vec{k}$ and the x direction. *α* is the angle between the plane of linear polarization and the x direction. Considering the contribution of asymmetric relaxation of electrons to the current, we should add an additional term to the $\rho_{\text{cv},\phi }^{\left(0\right)}$. Then the $\rho_{\text{cv},\phi }^{\left(0\right)}$ in Equation 6 includes contributions of both excitation and relaxation. Owing to the magneto-photocurrent in this superlattice is independent of the radiation polarization, it can be deduced that $\rho_{\text{cv},\phi }^{\left(0\right)}$ is much larger than $\rho_{\text{cv},\phi }^{\left(1\right)}$ and $\rho_{\text{cv},\phi }^{\left(2\right)}$. This conclusion is similar to that in [22] which that reported $\rho_{\text{cv},\phi }^{\left(0\right)}$ always overwhelms $\rho_{\text{cv},\phi }^{\left(1\right)}$ and $\rho_{\text{cv},\phi }^{\left(2\right)}$ theoretically.

The radiation polarization independent of MPE generated by direct interband transition had also been observed in the BiTeI film [23]. However, in (110)-grown GaAs/Al _*x*_*Ga*_1−*x*_ As quantum wells, MPE generated by indirect intrasubband transition shows clear relations to the radiation linear polarization state [24]. The reason may be that in the intrasubband transition process, spin-dependent asymmetric electron-phonon interaction which contributes to the magneto-photocurrent is sensitive to the radiation polarization state. It leads to the relative magnitudes of $\rho_{\text{cv},\phi }^{\left(1\right)}$ and $\rho_{\text{cv},\phi }^{\left(2\right)}$ in Equation 6 increase. More practically, the phonon effect may be taken into account when designing optically manipulated spintronics devices in the future.

**Figure 3:**
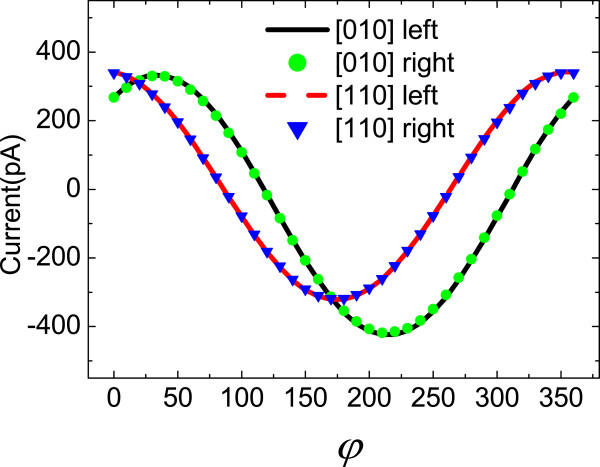
**The magneto-photocurrents in [010] and [110] crystallographic directions.** The black solid line and red dashed line denote currents excited by the left circularly polarized light. The green dots and blue inverted triangles denote currents excited by the right circularly polarized light. *φ* is the angle between the magnetic field direction and [1$\bar{1}$0] crystallographic direction

To research the magneto-photocurrents excited by circularly polarized light *via* rotating the in-plane magnetic field, we used the quarter-wave plate to obtain circularly polarized light. A 1,064-nm laser along -z was also used. The laser power was about 100 mW. As shown in Figure 3, the magneto-photocurrents under left and right circularly polarized light are nearly the same. It means that the circularly polarized light-dependent currents are vanishingly small compared to unpolarized light-dependent currents. Since the left and right circularly polarized light correspond to *P*_circ_=1 and −1 respectively, if the currents are circularly polarized light-sensitive, the waveform of the total currents would be obviously different in the two conditions. From the microscopic perspective, asymmetric spin-flip scattering mechanism of electrons which induces the spin-galvanic effect (SGE) [25] rarely contributes to the total magneto-photocurrents.

**Figure 4:**
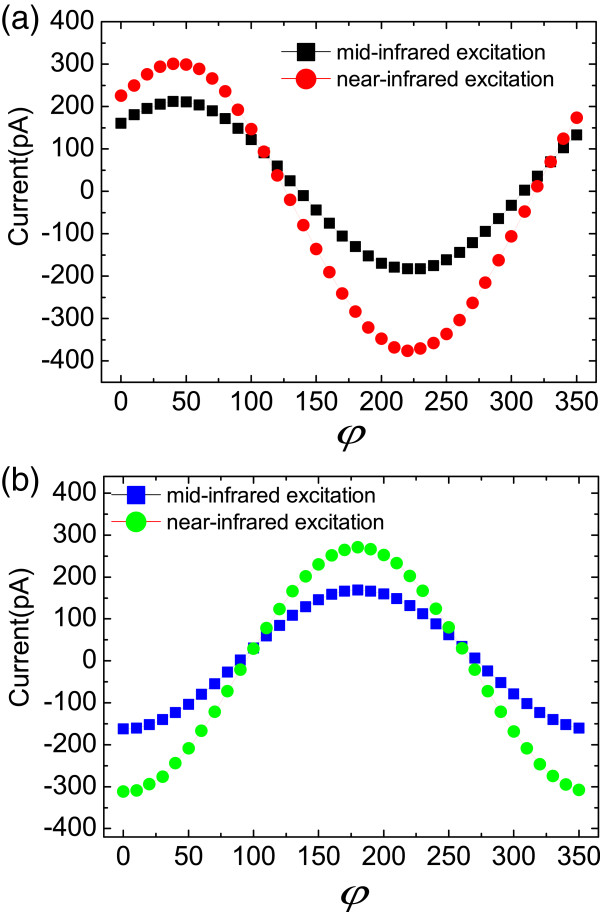
**The magneto-photocurrents in the (a) [010] crystallographic and (b) [110] directions. (a)** The black squares and red circles denote currents excited by mid-infrared radiation and near-infrared radiation, respectively. **(b)** The blue squares and green circles denote currents excited by mid-infrared radiation and near-infrared radiation respectively. *φ* is the angle between the magnetic field direction and [1$\bar{1}$0] crystallographic direction.

In the above, we have discussed the magneto-photocurrents in the InAs/GaSb superlattice generated by direct interband transition. Here, we present the results of magneto-photocurrents generated by intersubband transition for comparison. We utilized a CO _2_ continuous wave laser which can generate the mid-infrared radiation at 10.26 *μ*m (121.15 meV). The power of the excitation was approximately 60 mW and the linearly polarized direction was along [110] crystallographic direction. By rotating the magnetic field in the *x-y* plane, we obtained the dependence of the photocurrents on the magnetic field direction. As shown in Figure 4, in both [010] and [110] crystallographic directions, the waveform of the mid-infrared radiation-excited currents is similar to that of the near-infrared radiation-excited currents. The current curves share the identical phases in the two excitation conditions. That is for the mid-infrared excitation case, the currents also reach the maximum when the magnetic field is perpendicular to the detected direction and go to the minimum when the magnetic field is paralleled to the detected direction. It indicates that the unpolarized radiation-related current is dominant in the total magneto-photocurrents. In summary, for both the interband and intersubband excitation, the magneto-photocurrents are insensitive to the polarization state of the radiation. In another hand, we analyzed the peak-to-peak values of the currents (*J*_pp_) in the two excitation conditions. In the [010] crystallographic direction, the ratio of *J*_pp_ under mid-infrared radiation excitation to *J*_pp_ under near-infrared radiation excitation is 0.58. In the [110] crystallographic direction, the ratio is 0.57. The magnetic field-induced current conversion efficiency for near-infrared excitation is nearly twice as that for mid-infrared excitation.

### Tilted magnetic field-dependent MPE

**Figure 5:**
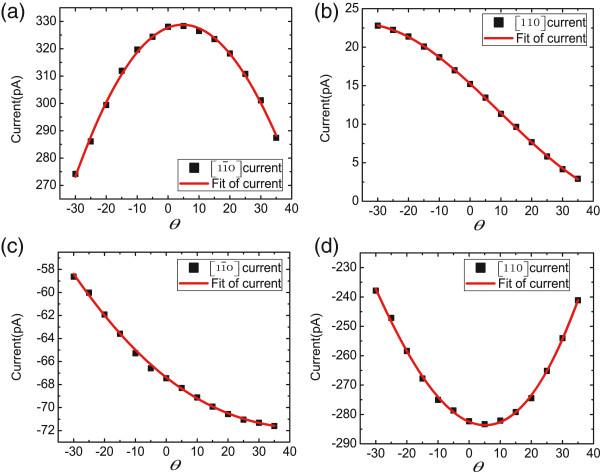
**Magneto-photocurrents in two crystallographic directions when magnetic field is rotated in (a,b) *y-z * and (c,d) *x-z * planes.** The red lines are the fitting curves of the currents in [1$\bar{1}$0] and [110] crystallographic directions. *θ* is the angle between the magnetic field direction and the sample plane.

In this section, we present results of a study of the magneto-photocurrents vs. the tilt angle of the magnetic field with respect to the sample surface. A linearly polarized 1,064-nm laser along -z was also used. The laser power was about 57 mW. The radiation linearly polarized direction was along the [100] and [010] crystallographic directions respectively when the magnetic field was rotated in the *y-z* and *x-z* planes. When the magnetic field is in the *y-z* plane, *B*_*y*_=*B*_0_ cos(*θ*), *B*_*z*_=*B*_0_ sin(*θ*) and *B*_*x*_=0. *θ* is the angle between the magnetic field direction and the sample plane. The experimental results are presented in Figure 5.

As shown in Figure 5, the photocurrents are well fitted by linear combination of sin2*θ*, sin*θ* and cos*θ* rather than by Equations 1 and 2. Thus, the mechanism of linear in-plane magnetic field-induced photocurrents (described by Equations 1 and 2) cannot hold here. Besides, the photocurrents cannot be explained by the mechanism of interplay of spin and orbit MPE observed in InSb/(Al,In)Sb quantum wells, [21] because the magnetic field strength here is too small. Nevertheless, we can use a model which combines linear in-plane magnetic field-dependent photocurrents and Hall effect [26]. A moderate in-plane magnetic field can induce photocurrents linearly proportional to the magnetic field strength in both x and y directions. These currents can be described by Equations 1 and 2. When the magnetic field is tilted, the z component of the magnetic field imposes Lorentz force on the electrons; therefore, part of electrons originally moving in the y direction bend to the x direction and vice versa. Thus, the total photocurrents superposed by the in-plane magnetic field-dependent photocurrent and the Hall effect-dependent current present quadratic magnetic field dependence. They can be described by Equations 7 and 8 when the magnetic field is in the *y-z* plane.

(7) $$\begin{array}{lll} J_{x} & =S_{1}IB_{0}\cos(\theta )+\frac{1}{2}\varepsilon_{y1}{S_{3}}^{\prime }I{B_{0}}^{2}\sin(2\theta )\; & \;\\ & \;+\varepsilon_{y2}C_{y}B_{0}\sin(\theta )+C_{x}.\; \end{array}$$

(8) $$\begin{array}{lll} J_{y} & ={S_{3}}^{\prime }IB_{0}\cos(\theta )+\frac{1}{2}\varepsilon_{x1}S_{1}I{B_{0}}^{2}\sin(2\theta )\; & \;\\ & \;+\varepsilon_{x2}C_{x}B_{0}\sin(\theta )+C_{y}.\; \end{array}$$

*ε*_*x* *i*_ and *ε*_*y* *i*_ are mixing parameters due to the Hall effect. *C*_*x*_ and *C*_*y*_ are background photocurrents. Whenthe magnetic field is in the *x-z* plane, the total currents can be described by

(9) $$\begin{array}{lll} J_{x} & =(-S_{3})IB_{0}\cos(\theta )+\frac{1}{2}{\varepsilon_{y1}}^{\prime }{S_{1}}^{\prime }I{B_{0}}^{2}\sin(2\theta )\; & \;\\ & \;+{\varepsilon_{y2}}^{\prime }{C_{y}}^{\prime }B_{0}\sin(\theta )+{C_{x}}^{\prime }.\; \end{array}$$

(10) $$\begin{array}{lll} J_{y} & ={S_{1}}^{\prime }IB_{0}\cos(\theta )+\frac{1}{2}{\varepsilon_{x1}}^{\prime }(-S_{3})I{B_{0}}^{2}\sin(2\theta )\; & \;\\ & \;+{\varepsilon_{x2}}^{\prime }{C_{x}}^{\prime }B_{0}\sin(\theta )+{C_{y}}^{\prime }.\; \end{array}$$

*ε*_*x* *i*_^′^ and *ε*_*y* *i*_^′^ are also mixing parameters due to the Hall effect. *C*_*x*_^′^ and *C*_*y*_^′^ are background photocurrents.

To fit the curves by Equations 7 and 10, we obtained the parameters *S*_1_ and *S*_1_^′^. The relations of parameters *S*_1_, *S*_1_^′^ getting from the in-plane and tilted magnetic field experimental configurations are shown in

(11) $$\begin{array}{lll} & {S_{1}}_{\text{(in)}}/{S_{1}}_{\text{(tilted)}}=0.91\; & \;\\ & {S_{1}}_{\text{(in)}}^{\prime }/{S_{1}}_{\text{(tilted)}}^{\prime }=0.96\; \end{array}$$

**Figure 6:**
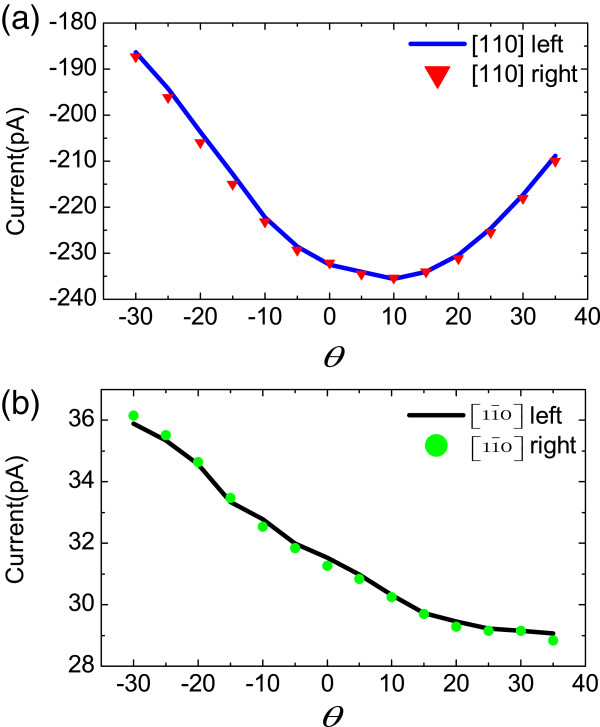
**The magneto-photocurrents in (a) [110] and (b) [1**$\bar{1}$**0] crystallographic directions.(a)** The blue solid line and red inverted triangles denote currents excited by left and right circularly polarized light, respectively. **(b)** The black solid line and green dots denote currents excited by left and right circularly polarized light, respectively. *θ* is the angle between the magnetic field direction and the sample plane.

Subscripts *in* and *tilted* signify parameters fitted from the in-plane and tilted magnetic field experiments, respectively. As shown in Equation 11, the parameters of the two configurations are nearly the same. This demonstrates that the theoretical model used in the tilted magnetic field experiments is reasonable. Besides, *S*_1_ and *S*_1_^′^ are much larger than *S*_3_ and *S*_3_^′^. It demonstrates that the magneto-photocurrents are also linear polarization-insensitive for the tilted magnetic field case. Figure 6 shows the magneto-photocurrents excited by circularly polarized light when the magnetic field is rotated in the *x-z* plane. In this case, a circularly polarized 1,064-nm laser along -z was used. The laser power was about 58 mW. As shown by the coincidence of the data from two different circular polarizations in Figure 6a,b, the experiments show that the currents are unrelated to the circular polarization state of the radiation.

In another hand, we presented the results of the magneto-photocurrents vs. the strength of magnetic field for comparison. A linearly polarized 1,064-nm laser, whose linearly polarized direction was along [110] crystallographic direction, was normally irradiated on the sample plane. The laser power was about 62 mW. The variable magnetic field generated by an electromagnetic device was in the *x-z* plane. The angle between the magnetic field and the sample plane was 12.5°. At a certain magnetic field, the magneto-photocurrents can be well described by Equations 9 and 10. However, these currents are superpositions of linear magnetic field and quadratic magnetic field-induced currents. To extract the pure quadratic magnetic field-dependent photocurrents, we eliminated the linear magnetic field-dependent currents by

(12) $$\begin{array}{l} J_{q}=\left[J(B)+J(-B)\right]/2 \end{array}$$

**Figure 7:**
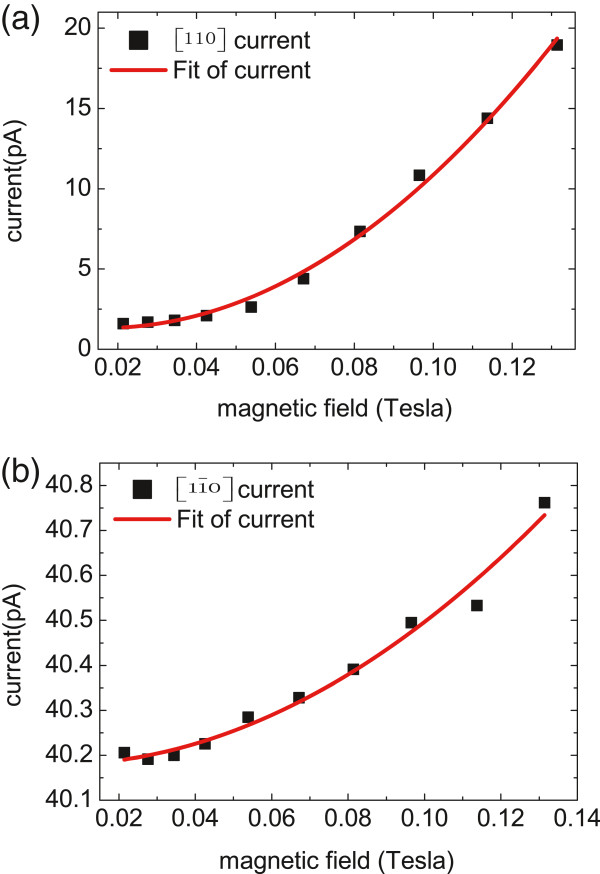
**The magneto-photocurrents *J***_***q***_** in (a) [110] and (b) [1**$\bar{1}$**0] crystallographic directions.** The red parabolic-shape lines are fitting curves of the currents.

The dependences of *J*_*q*_ on the strength of magnetic field are shown in Figure 7. We can see that the experimental data points are mainly in accord with the parabolic-shape fitting curves. The currents *J*_*q*_ presented clear quadratic magnetic field dependence. When the magnetic field was increased to 0.13 T, the current in [110] crystallographic direction increased by 17.35 pA; however, the current in [1$\bar{1}$0] crystallographic direction only increased by 0.56 pA. With the increase of the magnetic field, the current amplitude in [110] crystallographic direction increased faster than that in [1$\bar{1}$0] crystallographic direction, despite the magnetic field-independent background current in Figure 7b.

## Conclusions

In summary, we have researched magneto-photocurrents in the InAs/GaSb superlattice when an in-plane and tilted magnetic field were applied respectively. The magneto-photocurrents in both conditions are insensitive to the polarization state of the incident light. A theoretical model involving anisotropic photo-excited carriers density is utilized to explain the in-plane magnetic field-induced MPE. Compared to the direct electron-photon interaction, the asymmetric electron-phonon interaction which contributes to the magneto-photocurrent may be more sensitive to the radiation polarization state. The quadratic magnetic field dependence of the magneto-photocurrents can be well illustrated by an additional Hall effect model.

## Competing interests

The authors declare that they have no competing interests.

## Authors’ contributions

Y Li designed and carried out the experiments and wrote the manuscript. Y Liu and YC revised the paper. CJ, LZ, XQ and HG participated in the experiments. WM, XG and YZ designed and provided the sample. All authors read and approved the final manuscript.
